# Peripheral Giant Cell Granuloma Associated With Severe Periodontal Destruction in the Esthetic Region: A Case Report

**DOI:** 10.1155/crid/4055356

**Published:** 2026-05-30

**Authors:** Kubra Burcu Yildirim, Hanzade Hazal Sayiner

**Affiliations:** ^1^ Nova Southeastern University College of Dental Medicine, Fort Lauderdale, Florida, USA; ^2^ KEM Dental Clinic, Istanbul, Türkiye

**Keywords:** alveolar bone loss, case report, gingival lesion, periodontitis, peripheral giant cell granuloma, tooth mobility

## Abstract

Peripheral giant cell granuloma (PGCG) is a reactive lesion typically confined to the gingiva or alveolar mucosa and is generally regarded as a soft tissue entity. Although superficial bone involvement has occasionally been described, extensive periodontal destruction is not consistently observed. This report describes a case of PGCG occurring in the maxillary anterior region in association with advanced periodontal bone loss. A 37‐year‐old male presented with a progressively enlarging, asymptomatic swelling between Teeth #11 and #12 of 3 months′ duration. Clinical examination revealed a pedunculated, purple‐red mass measuring approximately 14 × 11 mm. Periodontal assessment demonstrated Stage III, Grade C periodontitis with deep probing depths extending toward the apical third of the roots and Grade III mobility of the involved teeth. Radiographic evaluation showed advanced horizontal bone loss and a pronounced interdental periodontal defect. The lesion was completely excised with adequate margins, and due to extensive periodontal support loss, Teeth #11 and #12 were extracted during the same surgical procedure. Histopathological analysis confirmed the diagnosis of PGCG. Healing was uneventful, and no clinical signs of recurrence were observed at 1‐month follow‐up. This case highlights that PGCG may coexist with advanced periodontal destruction in natural dentition and emphasizes the importance of comprehensive periodontal evaluation and complete surgical excision to minimize recurrence risk.

## 1. Introduction

Peripheral giant cell granuloma (PGCG) is among the most frequently encountered giant cell lesions of the oral cavity, arising exclusively within the gingival and alveolar mucosal tissues. [1]. It is generally regarded as a benign proliferative soft tissue growth. Most authors consider it to arise in response to chronic local irritation or minor trauma, with a probable origin from the periosteum or periodontal ligament [1, 2]. Plaque accumulation, calculus, food impaction, persistent inflammation, and defective restorations have all been implicated as contributing factors [2, 3]. Orthodontic therapy has also been suggested in some cases [4].

The mandible is affected more often than the maxilla, with a predilection for the interdental and interproximal areas [2, 5]. Clinically, it presents as a red to reddish‐purple nodular enlargement, which may be sessile or pedunculated [1]. The lesion mainly affects soft tissues. However, superficial “cupping” resorption of the underlying alveolar bone has been described [1, 2]. In most patients, bone involvement remains limited. Occasionally, more pronounced periodontal destruction and increased tooth mobility have been observed [6, 7].

In the present report, we describe a case of PGCG located in the maxillary anterior region and associated with advanced alveolar bone loss and marked mobility of the involved teeth. The clinical, radiographic, and histopathological findings are presented and discussed in relation to previously published data.

## 2. Case Presentation

A 37‐year‐old male was referred to our clinic because of a swelling in the maxillary anterior region. The patient reported that the swelling had first appeared approximately 3 months earlier and had progressively enlarged (Figure 1). He denied any prior lesion in the same region. The lesion was asymptomatic; however, the patient noted that following the increase in size, a visible spacing had developed between the adjacent teeth and that the involved teeth had gradually become mobile. There was no history of trauma in the area. His medical history was unremarkable; he was in good general health and taking no medications. The patient reported that he is a nonsmoker and had never smoked cigarettes but acknowledged regular hookah use.

**Figure 1:**
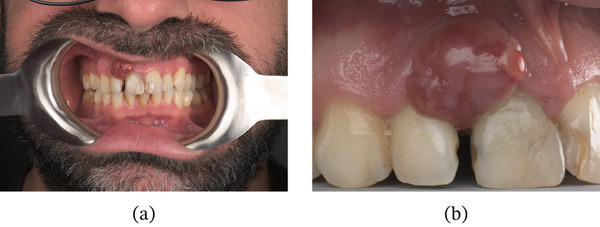
(a) Frontal intraoral view demonstrating the lesion in the maxillary anterior region. Note the generalized plaque accumulation and the diastema between Teeth #11 and #12 associated with the presence of the mass. (b) Close‐up view of the lesion showing a purple–red, lobulated, pedunculated mass located in the interdental region between Teeth #11 and #12, measuring approximately 14 × 11 mm.

Intraoral examination revealed a pedunculated exophytic mass located between Teeth #11 and #12. The lesion measured approximately 14 × 11 mm. It appeared purple–red in color and had a lobulated surface. The lesion was firm and slightly tender on palpation. No surface ulceration was observed.

Periodontal examination revealed poor oral hygiene, with full‐mouth plaque and bleeding scores exceeding 80%. Generalized attachment loss consistent with Stage III, Grade C periodontitis was observed throughout the dentition. Teeth #11 and #12 demonstrated Grade III mobility. In the affected region, probing depths extended close to the apical third of the roots. Clinically, the interdental tissues appeared inflamed, with bleeding on gentle probing.

Radiographic evaluation revealed generalized horizontal alveolar bone loss affecting the entire dentition. In the maxillary anterior region, periapical radiographs demonstrated more pronounced involvement, with advanced bone loss between Teeth #11 and #12 and a distinct radiolucency extending apically along the root surfaces, corresponding clinically to a probing depth of approximately 11 mm. Bone destruction extended nearly to the apical third of the lateral incisor root, consistent with the severity of clinical attachment loss and Grade III mobility observed during examination (Figure 2a,b).

**Figure 2:**
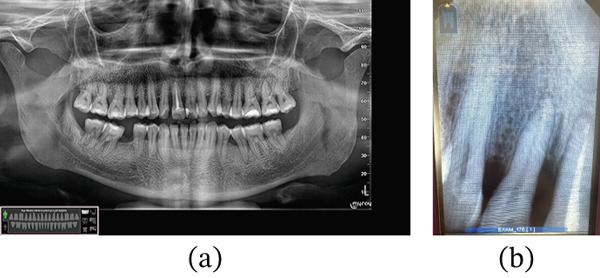
Radiographic evaluation of the maxillary anterior region. (a) Panoramic radiograph demonstrating generalized horizontal alveolar bone loss throughout the dentition, more pronounced in the anterior region. (b) Periapical radiograph of the maxillary anterior region. Teeth #11, #12, and #13 are indicated. Marked bone loss is evident between Teeth #11 and #12, extending apically along the root surface of the lateral incisor.

At this stage, the lesion was clinically suggestive of a reactive gingival growth. PGCG was strongly considered given the purple–red color, firm consistency, and relatively limited bleeding tendency of the mass. Pyogenic granuloma was included in the differential; however, it was considered less likely, as the lesion demonstrated a firmer consistency and bled only upon deep probing rather than on superficial contact, features more consistent with PGCG than with the typically friable and highly vascular pyogenic granuloma. Peripheral ossifying fibroma was also considered. A central giant cell granuloma was considered less likely because the lesion appeared confined to the soft tissues. Definitive diagnosis required complete surgical excision and histopathological evaluation.

Surgery was performed under local anesthesia. The lesion was excised with adequate margins, including its base. A sulcular incision was made around the involved teeth, and a full‐thickness mucoperiosteal flap was elevated to facilitate complete excision of the lesion and to allow thorough curettage of residual pathologic soft tissue and bone following removal. The mass was found to be firmly adherent to the periosteal surface and was removed in toto. The specimen was immediately fixed in 10% buffered formalin.

After excision, the underlying bone was carefully inspected. No gross bony perforation was observed. However, advanced horizontal bone loss and a deep intrabony periodontal component were confirmed. Thorough curettage of the adjacent bone and surrounding soft tissues was performed to eliminate residual pathologic tissue.

Given the severe loss of periodontal support and Grade III mobility, Teeth #11 and #12 were extracted during the same procedure. To obtain primary closure while preserving the existing keratinized tissue, periosteal releasing incisions were performed. This allowed passive apical repositioning of the flap. The flap was stabilized with interrupted sutures to achieve tension‐free adaptation and maintain the mucogingival architecture (Figure 3a–f).

**Figure 3:**
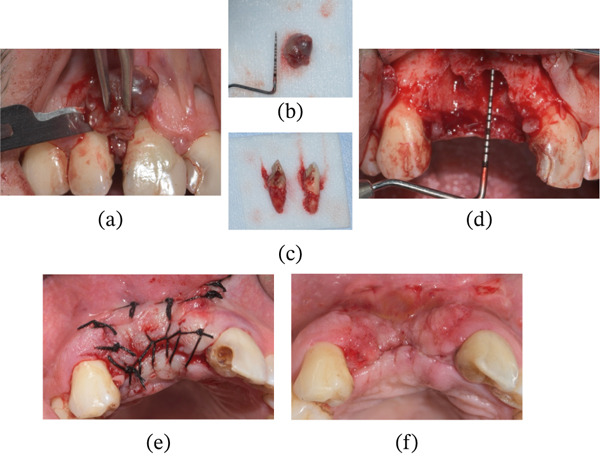
(a–f) Surgical procedure and intraoperative findings. (a) Excision of the lesion using a scalpel. (b) Excised lesion measured extraorally using a calibrated periodontal probe. (c) Extracted teeth (#11 and #12) following removal due to advanced periodontal destruction. (d) Intraoperative view following flap elevation, demonstrating the vertical alveolar bone defect probed to its full depth. (e) Postsuturing occlusal view showing tension‐free primary closure. (f) Clinical view at 10 days postoperatively, following suture removal, demonstrating satisfactory soft tissue healing.

The excised specimen was routinely processed and stained with hematoxylin and eosin for light microscopic evaluation.

Microscopic examination revealed a nonencapsulated inflammatory lesion composed of a fibroangiomatous stroma with areas of hemorrhage and hemosiderin deposition. Numerous osteoclast‐type multinucleated giant cells were observed within the lesion. A mixed inflammatory infiltrate consisting of lymphoplasmacytic and polymorphonuclear leukocytes was also present. No evidence of malignancy was identified.

The histopathological findings were consistent with a diagnosis of PGCG (Figure 4a–c).

**Figure 4:**
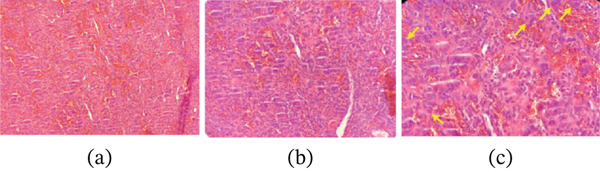
Histopathological examination of the excised lesion. (a) Low‐power view demonstrating the overall architecture of the lesion with vascularized stroma and areas of hemorrhage. (b) Medium‐power view showing the fibrovascular stroma with extravasated erythrocytes. (c) Higher power detail (H&E, original magnification ×200; digitally magnified for presentation) highlighting multinucleated giant cells (yellow arrows) within the lesion, characteristic of peripheral giant cell granuloma.

Healing progressed without complications. At the 1‐month follow‐up visit, satisfactory soft tissue healing was observed, and no clinical signs of recurrence were detected.

The patient expressed satisfaction with the surgical treatment and healing outcome. He reported an improvement in comfort and was relieved after the removal of the lesion and the extraction of the severely mobile teeth.

For esthetic and functional reasons, a provisional fixed partial prosthesis involving the maxillary anterior segment was delivered following initial healing.

This case report was prepared in accordance with the CARE guidelines, and the CARE checklist is provided as supporting information.

## 3. Discussion

PGCG is traditionally classified as a reactive lesion of the gingiva or alveolar mucosa, and according to the current WHO 2022 classification of head and neck tumors, it is defined as an osteoclast‐containing gingival/alveolar lesion originating from the periosteum or periodontal ligament in response to local irritating factors [8]. In most reported cases, the lesion follows an indolent clinical course with limited impact on the surrounding periodontal structures [2]. The present case is notable for its departure from this typical presentation: a PGCG arising in the maxillary anterior region in a patient with Stage III, Grade C periodontitis, associated with advanced alveolar bone loss, Grade III tooth mobility, and ultimately requiring extraction of the involved teeth. No prior report, to our knowledge, has documented this combination—PGCG coexisting with severe generalized periodontitis leading to tooth extraction in the anterior maxilla.

### 3.1. PGCG and Periodontitis: A Review of the Literature

The association between PGCG and periodontal tissue destruction has been described in a limited number of case reports. Moreno Rodríguez reported a case of PGCG associated with a two‐wall intrabony defect in a periodontally healthy patient, where complete defect resolution was achieved following excision and a targeted surgical protocol at 2‐year follow‐up [7]. More recently, Cuozzo et al. described a PGCG in a 12‐year‐old patient with buccal alveolar bone resorption and a probing depth of 11 mm, demonstrating complete periodontal regeneration over an 8‐year follow‐up period [9]. Critically, both of these cases describe PGCG‐associated periodontal destruction in the absence of generalized periodontitis, and the involved teeth were successfully preserved following surgical treatment.

The present case stands in marked contrast to these previously reported cases. The periodontal breakdown observed was not confined to the region of the lesion but reflected a generalized pattern of Stage III, Grade C periodontitis with full‐mouth plaque and bleeding scores exceeding 80%. The severe attachment loss and Grade III mobility of Teeth #11 and #12 extended beyond what could be attributed to a reactive lesion alone. The coexistence of PGCG and advanced generalized periodontitis in this case suggests that these two conditions may interact, producing a degree of tissue destruction that neither condition alone would be expected to generate, though the directionality of this relationship cannot be determined from a single case report.

### 3.2. Bone Resorption in PGCG: Extent and Mechanisms

Although superficial “cupping” resorption of the underlying alveolar bone has been described in some cases of PGCG, extensive periodontal destruction is not a consistently observed feature [1, 2]. The multinucleated giant cells of PGCG display the phenotypic and functional characteristics of osteoclasts, expressing tartrate‐resistant acid phosphatase, cathepsin K, matrix metalloproteinase‐9 (MMP‐9), RANK, and the calcitonin receptor [10, 11]. Despite this osteoclastic profile, radiographic osteolysis is absent in a significant proportion of PGCG cases. Friedrich et al. proposed that this apparent paradox is explained by the topography of the lesion relative to the adjacent bone: Multinucleated giant cells located distantly from the bone surface retain cathepsin K largely in its inactive precursor form, whereas those in close topographic proximity to bone express the mature, active enzyme capable of initiating bone matrix degradation [11]. This topography‐dependent activation model implies that the osteolytic potential of PGCG is not fixed but is determined in part by the anatomical relationship between the lesion and the underlying bone.

In the present case, the lesion was found to be firmly adherent to the periosteal surface at the time of surgical excision. This intimate periosteal contact, combined with the coexisting deep interdental bony defect, placed the lesion′s multinucleated giant cells in direct topographic proximity to bone—conditions that would be expected to favor cathepsin K activation and active bone resorption, regardless of which process initiated the defect.

### 3.3. Biological Mechanisms: A Proposed Framework for Amplified Bone Destruction

The molecular mechanisms underlying bone resorption in PGCG center on the RANKL/RANK/OPG axis. Stromal spindle‐shaped cells within the lesion express RANKL, which drives the differentiation and activation of osteoclastic multinucleated giant cells from monocyte/macrophage precursors [10, 12]. The osteoclastogenic cytokines TNF‐*α*, IL‐1*β*, and IL‐6, expressed by both the giant cells and stromal cells of PGCG, further stimulate this process both directly and by amplifying RANKL production by osteoblastic cells, while simultaneously suppressing osteoprotegerin—effectively shifting the RANKL/OPG balance in favor of bone resorption [12, 13].

In patients with periodontitis, an overlapping cytokine cascade operates through a distinct but convergent mechanism. Subgingival microbial biofilm and its associated virulence factors activate local macrophages and T lymphocytes, resulting in elevated local concentrations of TNF‐*α*, IL‐1*β*, and IL‐6 within the periodontal tissues, and the role of these cytokines in periodontitis‐associated alveolar bone loss is well established [13]. In the present patient, the high full‐mouth plaque and bleeding scores indicate that an active subgingival microbial environment was sustaining this proinflammatory cytokine milieu, particularly within the deep interdental pocket where the lesion was located.

We propose that the colocalization of PGCG within this periodontal inflammatory microenvironment may have contributed to amplified osteoclastic activity through two convergent mechanisms, though we acknowledge that this remains speculative in the absence of molecular data. First, the RANKL signals generated by the periodontitis‐driven host immune response and those generated by the PGCG stromal cells may have acted cumulatively at the same anatomical site, shifting the local RANKL/OPG ratio beyond what either process would have produced in isolation [13]. Second, chronic periodontal inflammation is associated with enzymatic fragmentation of high‐molecular‐weight hyaluronan (HMW‐HA) into low‐molecular‐weight fragments (LMW‐HA) by tissue hyaluronidases and matrix metalloproteinases. Under normal conditions, HMW‐HA binding to CD44 on osteoclast‐like cells downregulates MMP‐9 expression, thereby inhibiting osteoclast migration—a mechanism described as a physiological stop signal for bone‐resorbing cells [14]. Disruption of this regulatory mechanism by LMW‐HA fragments, as may occur in a chronically inflamed periodontal environment, could have further facilitated osteoclast migration and bone degradation at the site of the lesion [14, 15]. Whether these mechanisms were operative in the present case cannot be determined without direct molecular analysis, and their proposed role should be interpreted as a hypothesis warranting further investigation.

Taken together, the severity of bone destruction observed in this case may reflect the biological convergence of two processes sharing common osteoclastogenic mediators: the PGCG′s intrinsic osteoclastic machinery and the cytokine‐driven bone resorption of concurrent generalized periodontitis, both active within a shared anatomical site. This framework may offer a partial explanation for why PGCG in this case produced a degree of periodontal destruction far exceeding the superficial cupping resorption typically described in the literature, though the relative contribution of each process to the overall tissue loss cannot be quantified retrospectively.

### 3.4. Differential Diagnosis

Several reactive and neoplastic entities may present as exophytic gingival enlargements in the anterior maxilla, including pyogenic granuloma, peripheral ossifying fibroma, central giant cell granuloma, and irritation fibroma [1]. In this case, the purple–red color and bleeding tendency of the lesion initially raised the possibility of pyogenic granuloma; however, the firm consistency on palpation and the bleeding pattern—limited to deep probing rather than occurring on superficial contact—were more consistent with PGCG. Pyogenic granuloma typically presents as a highly vascular, friable mass that bleeds readily on minimal manipulation, a behavior not observed here. Central giant cell granuloma was considered unlikely given the soft tissue location of the lesion without primary intraosseous involvement. As with all gingival lesions of this nature, histopathological confirmation was indispensable for reaching a definitive diagnosis, confirming the presence of osteoclast‐type multinucleated giant cells within a fibroangiomatous stroma with hemosiderin deposits [2] (Table 1).

**Table 1:** Differential diagnosis of gingival soft tissue lesions.

Lesion	Clinical features	Radiographic findings	Histopathologic characteristics
Peripheral giant cell granuloma (PGCG)	Purple–red to bluish‐red pedunculated or sessile mass arising from the interdental papilla; firm on palpation; bleeding limited to deep probing	Superficial alveolar bone resorption may be present; extensive destruction is uncommon	Osteoclast‐type multinucleated giant cells distributed within a vascularized fibroblastic stroma; areas of hemorrhage and hemosiderin deposition
Pyogenic granuloma	Bright red, soft, friable exophytic mass; bleeds readily on minimal contact; commonly linked to local irritation, pregnancy, or hormonal fluctuation	No bone involvement in the majority of cases	Lobular proliferation of thin‐walled capillaries within a loose connective tissue stroma; multinucleated giant cells absent
Peripheral ossifying fibroma	Pink to red firm nodular mass arising from the interdental papilla; more prevalent in adolescents and young adults	Faint calcifications may be visible in mature lesions; cortical erosion is rare	Cellular fibrous stroma with foci of mineralization; may include woven bone, cementum‐like material, or dystrophic calcifications
Central giant cell granuloma	Intraosseous swelling with possible buccal or lingual cortical expansion; may cause root resorption or tooth displacement	Well‐demarcated unilocular or multilocular radiolucency within the jaw; no primary soft tissue origin	Multinucleated giant cells within a cellular fibrovascular stroma; the lesion arises centrally within the bone
Irritation fibroma	Pale pink, firm, smooth‐surfaced nodular mass; typically asymptomatic; associated with chronic mechanical irritation	No osseous involvement	Hypocellular dense collagenous connective tissue; absence of giant cells or significant vascularity

### 3.5. Surgical Management and Soft Tissue Considerations

Complete surgical excision, including the base of the lesion, remains the cornerstone of recurrence prevention, which has been reported at approximately 10% [2]. In this case, a full‐thickness mucoperiosteal flap was raised to facilitate complete excision of the lesion and to allow thorough curettage of residual pathologic soft tissue and bone following removal. Given the severity of attachment loss and Grade III mobility, extraction of Teeth #11 and #12 was performed during the same procedure.

Particular attention was given to soft tissue management in this esthetically sensitive region. Periosteal releasing incisions were performed to enable passive apical repositioning of the flap, with care taken to retain the residual keratinized gingiva. Achieving tension‐free primary closure was considered especially important given the patient′s relatively young age and the functional and esthetic significance of the maxillary anterior segment. In similar situations, future rehabilitative options may include vertical bone augmentation or regenerative approaches prior to implant placement; however, the potential for PGCG recurrence should be taken into account when planning definitive therapy, and staged treatment with a sufficient observation period is prudent prior to proceeding with advanced reconstructive treatment [2].

### 3.6. Follow‐Up and Limitations

Suture removal at 10 days revealed satisfactory healing with no wound complications. At the 1‐month visit, soft tissue healing was complete, and no clinical signs of recurrence were observed. At 3 months, the patient was contacted by telephone and reported no symptoms; however, this could not be corroborated by clinical examination. Given that recurrence has been documented in cases in which removal was incomplete or contributing local factors remained [2], continued long‐term monitoring remains advisable, especially in the present case, considering the severity of the underlying periodontal disease.

Several limitations should be acknowledged. No prior periodontal records were available, which prevented definitive determination of the temporal relationship between the PGCG and the surrounding bone loss. The mechanistic framework proposed in this discussion is therefore inferential, supported by radiographic and clinical findings rather than longitudinal or molecular data. Both the clinical presentation—generalized attachment loss and full‐mouth plaque and bleeding scores exceeding 80% throughout the dentition—and the radiographic findings—generalized horizontal alveolar bone loss affecting multiple quadrants on the panoramic image, well beyond the region of the lesion—are consistent with a longstanding periodontal disease process independent of the reactive lesion, though prospective follow‐up data would be needed to confirm this interpretation. Additionally, as a single case report, the observations presented here cannot be generalized, and the relative contribution of the PGCG versus the concurrent periodontitis to the overall tissue destruction remains impossible to quantify. These questions may be better addressed through dedicated prospective case series or through molecular characterization of the inflammatory microenvironment in PGCG lesions arising within periodontitis‐affected sites.

## 4. Conclusions

PGCG may present in association with advanced periodontal destruction in natural dentition and, in rare cases, may be linked to severe attachment loss requiring tooth extraction. Careful differential diagnosis, comprehensive periodontal assessment, and complete surgical excision with elimination of local contributing factors are essential to guide treatment and limit the risk of recurrence.

## Author Contributions

Kubra Burcu Yildirim had full access to all of the data in this study and takes complete responsibility for the integrity of the data and the accuracy of the data analysis. Kubra Burcu Yildirim contributed to the conceptualization, methodology, investigation, resources, data curation, writing—original draft, writing—review and editing, supervision, project administration, visualization, and validation. Hanzade Hazal Sayiner contributed to the investigation and writing—review and editing.

## Funding

No funding was received for this manuscript.

## Disclosure

All authors have read and approved the final version of the manuscript.

## Ethics Statement

Ethical approval was not required for this study, as it constitutes a single anonymized case report with written informed consent obtained from the patient.

## Consent

Written informed consent was obtained from the patient for the publication of this case report and accompanying images.

## Conflicts of Interest

The authors declare no conflicts of interest.

## Supporting information


**Supporting Information** Additional supporting information can be found online in the Supporting Information section. Supporting Information 1: CARE checklist for the case report titled “Peripheral Giant Cell Granuloma Associated With Severe Periodontal Destruction in the Esthetic Region: A Case Report.”

## Data Availability

All relevant data supporting the findings of this case report are included within the article.
